# Nightmares that mislead to diagnosis of reactivation of PTSD

**DOI:** 10.3402/ejpt.v4i0.18714

**Published:** 2013-04-01

**Authors:** Stefan Roepke, Marie-Luise Hansen, Anita Peter, Angela Merkl, Carla Palafox, Heidi Danker-Hopfe

**Affiliations:** Department of Psychiatry, Charité—Universitätsmedizin Berlin, Campus Benjamin Franklin, Berlin, Germany

**Keywords:** posttraumatic stress disorder, REM sleep behaviour disorder, nightmares, periodic limb movements, sleep disturbance

## Abstract

**Background:**

Sleep disturbance is a common characteristic of patients with post-traumatic stress disorder (PTSD). Besides the clinical descriptions of nightmares and insomnia, periodic limb movements (PLMs) are reported to co-occur in PTSD. Although the causal relationship between sleep disturbance and PTSD is not fully understood, sleep disturbance is an independent risk factor for the development and reactivation of PTSD. In contrast, the link between PTSD and REM sleep behaviour disorder (RBD) is less clear.

**Method:**

A case report is presented to illustrate differential diagnosis and time course of sleep disturbance in the context of PTSD.

**Result:**

A 63-year-old man who had been successfully treated for PTSD but who suffered the re-occurrence of disturbed sleep due to RBD and PLM, which was misdiagnosed as reactivation of PTSD.

**Conclusions:**

RBD can mimic PTSD-related nightmares. Accurate diagnosis of sleep disturbance in PTSD is relevant for treatment and prognostic evaluation.

Post-traumatic stress disorder (PTSD) is a psychiatric condition that can result from directly experiencing, witnessing, or learning about an actual or threatened traumatic event. PTSD patients show symptom clusters that can be grouped into three domains: 1) Intrusion symptoms (e.g., distressing dreams); 2) Avoidance of distressing memories and external reminders; and 3) Marked alterations in arousal and reactivity (e.g., difficulty falling or staying asleep or restless sleep) (DSM-IV, American Psychiatric Association [APA], 2000). PTSD is often unrecognised in general outpatient psychiatry (Al-Saffar, Borgå, & Hällström, 2002; Meltzer et al., 2012), even though it is related to low improvement rates and low general health compared to psychiatric patients without PTSD. In primary care settings, the situation is even worse; only 2–11% of cases with PTSD actually have the diagnosis noted in the medical record (Liebschutz et al., 2007; Taubman-Ben-Ari, Rabinowitz, Feldman, & Vaturi, 2001). In addition, less than half of these patients with PTSD actually receive treatment for PTSD (Rodriguez et al., 2003; Stein, McQuaid, Pedrelli, Lenox, & McCahill, 2000).

The clinical description clearly depicts the central role of sleep disturbance in PTSD. Indeed, approximately 70% of patients with PTSD report sleep disturbance, 41% of initiating and 47% of maintaining sleep (Ohayon & Shapiro, 2000). Furthermore, 50–70% of PTSD patients report recurrent nightmares (Kilpatrick et al., 1998; Leskin, Woodward, Young, & Sheikh, 2002; Neylan et al., 1998). Apart from those sleep disturbances listed in the clinical description of PTSD, periodic limb movements (PLMs) and sleep-disordered breathing (SDB) also often co-occur in PTSD patients: SDB has been found in 50–90% of PTSD patients in different study populations (Krakow et al., 2001, 2002, 2004), and clinically significant increases in PLMs have been found in different PTSD populations of veterans and sexual assault survivors, compared to the non-PTSD control groups (Brown & Boudewyns, 1996; Krakow et al., 2000; Mellman, Kulick-Bell, Ashlock, & Nolan, 1995; Ross et al., 1994). To date, only one study of veterans with a mid-50s mean age found an association between REM sleep behaviour disorder (RBD) and PTSD, although a common pathophysiology (i.e., locus coeruleus dysfunction) has been hypothesized (see Husain, Miller, & Carwile, 2001). However, a number of well-conducted polysomnographic studies of veterans could not replicate that finding (e.g., Capaldi, Guerrero, & Killgore, 2011; Germain et al., 2012; Van Liempt, 2012), which could be due to the age of participants or duration of the study of PTSD.

Accumulating evidence suggests that sleep disturbance is a risk factor for the development of PTSD following a traumatic event (Harvey & Bryant, 1998; Koren, Arnon, Lavie, & Klein, 2002; Mellman, David, Bustamante, Torres, & Fins, 2001). There is even some evidence that sleep disturbance, especially nightmares, was more prevalent in trauma victims who developed PTSD before trauma occurred (Mellman et al., 1995, Van Liempt, 2012). Sleep disturbances are also frequent residual symptoms after PTSD treatment (Zayfert & DeViva, 2004). Furthermore, persistence of sleep disturbances after PTSD treatment is a negative predictor for long-term outcome in different psychopathological and somatic domains (e.g., Belleville, Guay, & Marchand, 2011).

Clinical case reports (Christenson, Walker, Ross, & Maltbie, 1981; Russo, Hersen, & Van Hasselt, 2001) and larger sample studies (Macleod, 1994; Solomon, Garb, Bleich, & Grupper, 1987) have reported reactivation of PTSD in 11–19% of cases (Boe, Holgersen, & Holen, 2010; Port, Engdahl, & Frazier, 2001; Solomon & Mikulincer, 2006). Reactivation often occurs after exposure to new traumatic events (Haley, 1978), in relation to physical illness (Macleod, 1994), retirement (Port et al., 2001), and aging (Archibald & Tuddenham, 1965; Christenson et al., 1981; Heuft, 1999; Hiskey, Luckie, Davies, & Brewin, 2008). A predictor for later activation of PTSD seems to be the number of residual symptoms after PTSD remission, primarily intrusion symptoms, and avoidance (Boe et al., 2010). In particular, sleep-related intrusions, such as sleeping difficulties due to intrusive thoughts and bad dreams about the event, seem to have a strong predictive value for reactivation of PTSD (Boe et al., 2010).

To illustrate differential diagnosis and time course of sleep disturbance in the context of PTSD, we present a case of a patient in whom occurrence of sleep disturbance (nightmares) gave rise to suspected reactivation of PTSD.

## Case report

A 63-year-old man was admitted to a second round of trauma-focused psychotherapy with “severe nightmares” under the assumption that he was suffering from reactivation of PTSD. Ten years earlier, the patient had witnessed the murder of a close relative and had developed full PTSD with intrusions (including nightmares), avoidance behaviour, hyperarousal, and alterations in cognition and mood. The patient did not report any prior trauma. One year after the event, the patient already underwent 25 sessions of trauma-focused cognitive behavioural therapy (Ehlers, Clark, Hackmann, McManus, & Fennell, 2005) with full remission of the intrusions. Subsequent to this treatment, mild avoidance behaviour and mild hyperarousal persisted along with minor limitations of the activities of daily living. However, the patient's wife reported that, 1 year prior to admission, the patient had started screaming and moving excessively at night. This behaviour progressed and resulted in the use of separate bedrooms. On waking, the patient remembered undefined nightmares and restless sleep. Based on the patient's self-report, nightmares in the first PTSD episode (nine years before admission) were more vivid and trauma related. Six months prior to admission, a mild depressive syndrome and occasional mild flashbacks (e.g., seeing the dead body of his relative) reoccurred. Since 4 weeks prior to admission, the patient received quetiapine 25 mg per day at night. The medication did not influence subjective perception of sleep quality and nightmares. At admission, quetiapine medication was stopped without influence on clinical symptoms. The patient did not report any somatic illness at admission and vital parameters (blood pressure, pulse) and assessment of basic biomarkers revealed no pathological findings (electrolytes, liver-enzymes, creatinine, blood count, and inflammation markers). The SCID I interview (First et al., 1996) was performed by an experienced psychologist. The patient did not fulfil PTSD criteria, as hyperarousal and avoiding behaviour were not present, although the patient reported mild flash backs occasionally (once per week).

Due to the severe agitation at night (resulting in ecchymoses, lacerations, and the patient falling out of bed) a polysomnography (PSG) was performed. The PSG was recorded by a Nihon Koden Neurofax EEG 1200 G, in accordance with the requirements of the AASM recommendation (Iber, Ancoli-Israel, Chesson, Quan, & for the American Academy of Sleep Medicine, 2007). The scoring was performed by an experienced scorer according to AASM criteria B.

**Fig. 1:**
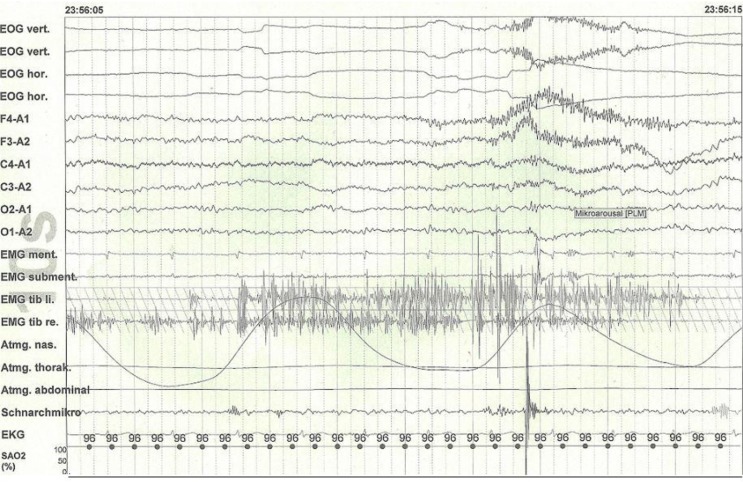
Example (10 seconds) of faulty regulation of motor activity in REM sleep indicating RBD. Note: The time line of Fig. 1 is 10 s. Legend top to bottom: EOG vert./hor.=electrooculogram—eye movements vertical or horizontal; F4, F3=frontal electrodes right and left; C4, C3=central electrodes right and left; O2, O1=occipital electrodes right and left; EMG electromyogram=muscle activity; ment. subment.=mental, submental; EMG tib li, tib re.=left and right muscle activity of lower leg (mm. tibiali); Atmg.=breathing; nas.=nasal; thorak.=thoracic; Schnarchmikro=snoring; EKG=ECG; SAO2=O2-saturation. Note the beginning of muscle activity in the form of twitches, moving to almost continuous muscle activity, first in the right m. tibialis anterior, then spreading to the left one, too. On the EOG, rapid eye movements are clearly visible, indicating ongoing REM. In the last third of the episode some muscle activity spreads to head muscles as well. No muscle activity in mental and submental muscles during the whole event; that is, these muscles maintained REM-atonia.

Healthy sleepers normally show a loss of muscle tone in REM sleep. But here in the PSG, the lack of muscle atonia during REM sleep, as indicated by merging muscle twitches in the m. tibialis during rapid eye movements in the EOG, establishes the diagnosis of RBD (for review, see Arnulf, 2012). As is typical in RBD, the muscle activity is not continuous, but consists of single twitches with a duration of 70–100 ms (note the single twitches in the first 3 seconds of EMG in the right m. tibialis in Fig. 1) which increase in frequency until merging into near-continuous muscle activity. In this example (see Fig. 1), the muscle activity spreads to the mm. frontales (F3-A1, F4-A2 and EOG vert. in the 7th to 9th second) and ends in a microarousal.

**Fig. 2:**
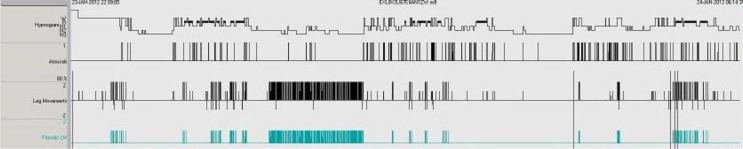
PSG results before initiating clonazepame and pramipexole treatment. Note: from top to bottom: 1, hypnogram showing the NREM-REM-cycles and interruptions thereof; 2, display of arousals; 3, overall leg movements; 4, periodic leg movements. Notice heavy fragmentation of REM sleep periods, often correlated to periodic limb movements. In NREM there is a long cluster of PLMs.

Furthermore, the patient showed moderate PLM during sleep (see Fig. 2). A total number of 286 such PLMs were observed, mainly in a cluster during the first half of the night. Eighteen of these limb movements were associated with an arousal and, in most cases, minor movements such as lifting the head, grasping or jerking also occurred. During REM sleep three episodes were observed in which the patient experienced a leg movement followed by rapid movements of an arm or a hand. In one episode he even struck out and hit the bedside cabinet with a loud knock. Several examples of sequences of events in REM sleep were observed: snoring followed by leg movement followed by jerks of the body, raising of the head, fumbling or beating with hand, or vocalisation.

The patient showed no clinical signs of Parkinson's disease, dementia with Lewy bodies, or multiple system atrophy; that is, no rigor, tremor, akinesia, dementia, visual hallucination, decreased olfaction or colour vision, or orthostatic hypotension. A cranial MRI revealed a few non-specific white matter lesions, but the basal ganglia and brainstem were unaffected. A routine EEG revealed a normal alpha-rhythm with no epileptic discharges.

A pharmacotherapy with 0.5 mg of clonazepame and 0.18 mg of pramipexole at night was initiated (for a review of treatment strategies for sleep disorders, see Lamarche & De Koninck, 2007; Roepke & Ancoli-Israel, 2010) and led to the full clinical remission of sleep disturbances. In a re-evaluation 3 months after discharge, the patient's wife, who was once again sharing a bedroom with her husband, reported minor movements or vocalisations at night, mainly when the patient did not take the clonazepame medication, which occasionally occurred. Furthermore, symptoms of depression and intrusions remained in remission.

## Discussion

This case nicely illustrates that sleep disturbance in PTSD might have a variety of underlying causes, such as independent sleep-wake disorders. Accurate diagnosis of sleep disturbance, for example, by using PSG, is essential as specific treatment options for different sleep-wake disorders exist (Lamarche & De Koninck, 2007; Roepke & Ancoli-Israel, 2010).

With regard to PLM, one can only speculate about the time course of the disorder. PLM could represent a residual symptom of initial PTSD treatment, or it could occur progressively thereafter. Additionally, in this case, PSG indicates that PLM can trigger arousal and awakening in REM (see Fig. 2), in addition to arousals that occur through RBD itself. Thus, PLMs in sleep might further trigger RBD, a finding that needs to be addressed in future studies.

With regard to RBD, there is a close association between RBD, narcolepsy and neurodegenerative disorders, in particular α-synucleinopathies such as Parkinson's disease, multiple system atrophy, and dementia with Lewy bodies (Manni, Ratti, & Terzaghi, 2011). Prospective studies estimate that neurodegenerative disease will develop in 40–65% of idiopathic RBD cases and onset averages between 10–25 years (Ferini-Strambi et al., 2011; Postuma, Gagnon, & Montplaisir, 2012). Thus, regular clinical monitoring is recommended in patients with idiopathic RBD. Only one study (Husain et al., 2001) could find increased co-occurrence of PTSD and RBD, whereas a number of other studies failed (e.g., Capaldi et al., 2011; Germain et al., 2012; Van Liempt, 2012). Although we can only speculate about causality, RBD might, in this case, have developed independent of prior PTSD. Nevertheless, further studies are needed to explore the temporal and psychopathological association between PTSD and RBD.

## Conclusion

RBD should be considered as differential diagnosis to PTSD in the clinical context of nightmares and severe movements during sleep. Uncharacteristic or treatment-resistant sleep disturbance should be further analysed with polysomnography as it may be caused by a particular sleep-wake disorder for which specific treatments exist.
